# Platelet adhesion on commercially pure titanium plates in vitro I: effects of plasma components and involvement of the von Willebrand factor and fibronectin

**DOI:** 10.1186/s40729-019-0160-z

**Published:** 2019-02-25

**Authors:** Akira Takahashi, Shotaro Takahashi, Tetsuhiro Tsujino, Kazushige Isobe, Taisuke Watanabe, Yutaka Kitamura, Takao Watanabe, Koh Nakata, Tomoyuki Kawase

**Affiliations:** 1 Kawasaki, Japan; 2 Tokyo Plastic Dental Society, Kita-ku, Tokyo, Japan; 3 Division of Anatomy and Cell Biology of the Hard Tissue, Institute of Medicine and Dentistry, Niigata University, Niigata, Japan; 4 Department of Oral and Maxillofacial Surgery, Matsumoto Dental University, Shiojiri, Japan; 5 Department of Oral Science, Graduate School of Dentistry, Kanagawa Dental University, Yokosuka, Japan; 6 Bioscience Medical Research Center, Niigata University Medical and Dental Hospital, Niigata, Japan; 7 Division of Oral Bioengineering, Institute of Medicine and Dentistry, Niigata University, Niigata, Japan

**Keywords:** Platelet, Titanium, Adhesion, Fibronectin, von Willebrand factor, Fibrinogen

## Abstract

**Background:**

Platelet-rich plasma (PRP) is widely used in regenerative dentistry. Furthermore, it is often applied in the pretreatment of titanium implants to improve their surface bioaffinity and initial stability. However, effects of PRP application on implant surface at cellular and molecular levels remain poorly understood. Therefore, we examined platelet adhesion on commercially pure titanium (*cp*-Ti) plates, with a particular focus on fibrinogen (FGN), von Willebrand factor (vWF), and fibronectin (FN), in the presence or absence of plasma components.

**Methods:**

Citrated blood samples were obtained from six healthy male volunteers, and pure-PRP (P-PRP) and pure platelet suspensions in phosphate-buffered saline (PBS) were prepared. Platelet adhesion on *cp*-Ti plate surface was evaluated by phalloidin staining and tetrazolium dye assay. Distribution of FGN, vWF, FN, albumin, CD62P, and CD63 was examined by immunocytochemical analysis.

**Results:**

Platelets in PBS suspensions rapidly and time-dependently adhered to *cp*-Ti plate surface, but this adhesion was substantially disturbed by the presence of plasma components. FGN was most preferably adsorbed regardless of the presence or absence of plasma components, while vWF and FN showed greater accumulation on platelet adhesion area.

**Conclusions:**

Although FGN is rapidly and abundantly adsorbed on *cp*-Ti plate surface, vWF and FN function as major platelet adhesion molecules in citrated blood samples. After pretreatment with P-PRP, however, platelets adhered to *cp*-Ti much less efficiently. Therefore, P-PRP pretreatment might not directly contribute to surface functionalization, initial stabilization, and osseointegration of machined or similar types of implants.

**Electronic supplementary material:**

The online version of this article (10.1186/s40729-019-0160-z) contains supplementary material, which is available to authorized users.

## Introduction

Platelets rapidly accumulate at the sites of injury to prevent bleeding and repair damaged tissue and organs by releasing growth factors [1]. This fundamental function of platelets is the basis of regenerative therapy using platelet-rich plasma (PRP) [2]. However, platelets in PRP are considered mainly as growth factor carriers. To maximize the potential of PRP and expand its clinical applicability, clinicians must precisely understand functions of platelets in the form of PRP as well as analyze the quality of individual PRP preparations prior to clinical use.

PRP is often used in regenerative dentistry to acclimatize dental implant surface to in vivo conditions and, more specifically, to functionalize the surface to improve its bioaffinity [3–10]. The ultimate goal of this challenging application is to enhance the osseointegration of dental implants. Although upon implantation, dental implants probably immediately trigger coagulation and activate inflammatory reactions in vivo, there is no evidence regarding the mutual interactions between dental implants and citrated PRP in vitro. Additionally, the stability of PRP-coated implants in vivo remains unknown. Furthermore, the extent of platelet adhesion and adsorption of platelet adhesion molecules, along with platelet-derived growth factors and other plasma components, on implants surface remain unclear. To our knowledge, the use of PRP is not supported by scientific evidence to date, and its effect on implant stabilization and osseointegration remains controversial.

Given this, in this study, we established an experimental system using plain commercially pure titanium (*cp*-Ti) plates to examine platelet adhesion and adsorption of platelet adhesion molecules [fibrinogen (FGN), von Willebrand factor (vWF), and fibronectin (FN)] on implant surface in the presence and absence of plasma components. FGN was specifically and abundantly adsorbed, but vWF and FN were the major platelet adhesion molecules on *cp-*Ti plate surface both in the presence and absence of plasma components.

## Materials and methods

### Preparation of platelet suspensions in phosphate-buffered saline and plasma

Blood samples were collected from six non-smoking healthy male volunteers aged 23 to 58 years. The study design and consent forms for all procedures were approved by the ethics committee for human participants at the Niigata University School of Medicine (Niigata, Japan) and complied with the Helsinki Declaration of 1964, as revised in 2013.

First, ~ 9 mL of peripheral blood was collected in plain plastic vacuum blood collection tubes (Neotube; NIPRO, Osaka, Japan) containing 1 mL of A formulation of acid-citrate-dextrose (ACD-A; Terumo, Tokyo, Japan) [11, 12]. Whole-blood samples were stored in a rotating agitator at ambient temperature and were used within 48 h [13, 14]. Thereafter, the samples were centrifuged at 472×*g* for 10 min (soft spin). The upper plasma fraction, ~ 2 mm beyond the interface between the plasma and red blood cell fractions, was then transferred into 2-mL sample tubes, incubated with 5 μg/mL of prostaglandin E_1_ (PGE_1_; Cayman Chemical Co., Ann Arbor, MI, USA) for 10 min, and centrifuged again at 1065×*g* for 5 min (hard spin) to collect resting platelet pellets. Next, platelets were suspended in an appropriate volume of phosphate-buffered saline (PBS) or resuspended in the remaining supernatant (i.e., acellular plasma) after reduction of the volume to adjust the platelet concentration between 2.2 × 10^5^/μL and 2.8 × 10^5^/μL. Platelets and other blood cell counts were measured using a pocH 100iV automated hematology analyzer (Sysmex, Kobe, Japan). Leukocytes were not included; therefore, the platelet suspension in plasma was designated as “pure-PRP” (P-PRP).

### *cp*-Ti plates and platelet inoculation

*cp*-Ti plates (Nilaco, Tokyo, Japan) were cut into small, square 10 × 10 mm^2^ pieces; washed serially with acetone (60 s), ethanol (2 × 60 s), and distilled water (2 × 60 s) in an ultrasonic cleaner (Citizen, Tokyo, Japan), and air-dried.

Platelet suspensions, prepared as described earlier, were inoculated onto the *cp*-Ti plates, and the plates were incubated at ambient temperature for up to 60 min. The *cp*-Ti plates were then vigorously washed two times with PBS on a shaker (~ 10 s) and subjected to spectrophotometric assay without fixation or with 10% neutralized formalin fixation for staining. For activation, platelets in PBS suspensions were treated with 0.1% CaCl_2_, as described in previous studies [11, 12].

### Quantitative and qualitative determination of adherent platelet counts

Platelets were inoculated onto the surface of *cp*-Ti plates and incubated for 30 min. Then, the *cp*-Ti plates were washed with PBS to remove nonadhered platelets, as described earlier, and were further incubated for 2 h with a highly water-soluble tetrazolium dye (Cell Counting Kit-8; Dojindo, Kumamoto, Japan). After incubation, 100 μL of supernatant was collected, and its absorbance was measured at 450 nm.

Alternatively, platelets were fixed with 10% neutralized formalin, microperforated with 0.1% Tween-20-containing PBS (T-PBS) for 1 min, and stained with phalloidin (Cytopainter Phalloidin-iFlour 555 Reagent; Abcam, Cambridge, MA, USA) at ambient temperature in the dark and observed under a fluorescence microscope (ECLIPSE 80i; Nikon, Tokyo, Japan) connected with a cooled CCD camera (VB-7000; Keyence, Osaka, Japan).

### Immunocytochemical fluorescence staining

The *cp*-Ti plates were washed with PBS, and fixed platelets were microperforated with T-PBS for 1 min. The samples were washed twice with PBS and blocked with 0.1% Block ACE (Sumitomo Dainippon Pharma Co., Ltd., Osaka, Japan) in T-PBS for 1 h. Then, the samples were treated with mouse monoclonal anti-CD62P or anti-CD63 antibody (1:20 dilution; BioLegend, San Diego, CA, USA) for 60 min at ambient temperature; with rabbit polyclonal anti-fibrinogen (1:16 dilution; MBL, Nagoya, Japan), anti-fibronectin (1:200 dilution; Abcam), anti-vitronectin (1:100 dilution; Abcam), and anti-von Willebrand factor (1:200 dilution; Abcam); or with rabbit monoclonal anti-serum albumin (1:500 dilution; Abcam) overnight at 4 °C. Posttreatment, the samples were again washed twice with T-PBS and then probed with a secondary antibody (i.e., goat anti-mouse IgG H&L or anti-rabbit IgG H&L antibody conjugated with Alexa Fluor 488; Abcam) for 60 min along with phalloidin at ambient temperature in the dark.

Isotype controls for rabbit primary antibodies (Life Technologies Corporation, Carlsbad, CA, USA) and mouse primary antibodies (Abcam) were used as a negative control.

Finally, after a subsequent wash with PBS, the samples were mounted using an antifade mounting medium (Vectashield; Vector Laboratories, Burlingame, CA, USA), and target proteins were examined under a fluorescence microscope (ECLIPSE 80i; Nikon) connected with a cooled CCD camera (VB-7000; Keyence) [12].

### Sample preparation and AFM measurements

The *cp*-Ti plates with fixed platelets were washed three times with PBS and subjected to atomic force microscope (AFM), as described in previous studies [11, 15]. AFM height images of the samples were recorded in alternating current (AC) mode at room temperature in PBS using the NanoWizard 3 (JPK Instruments AG, Berlin, Germany) AFM system. Soft cantilevers (Tap300-G; BudgetSensors, Sofia, Bulgaria) were used for scanning.

### Statistical analysis

Data were expressed as mean ± standard deviation (SD). For two-group comparisons, Student’s *t* test was used to compare mean values (SigmaPlot 12.5; Systat Software, Inc., San Jose, CA, USA). *P* < 0.05 was considered statistically significant.

## Results

**Fig. 1:**
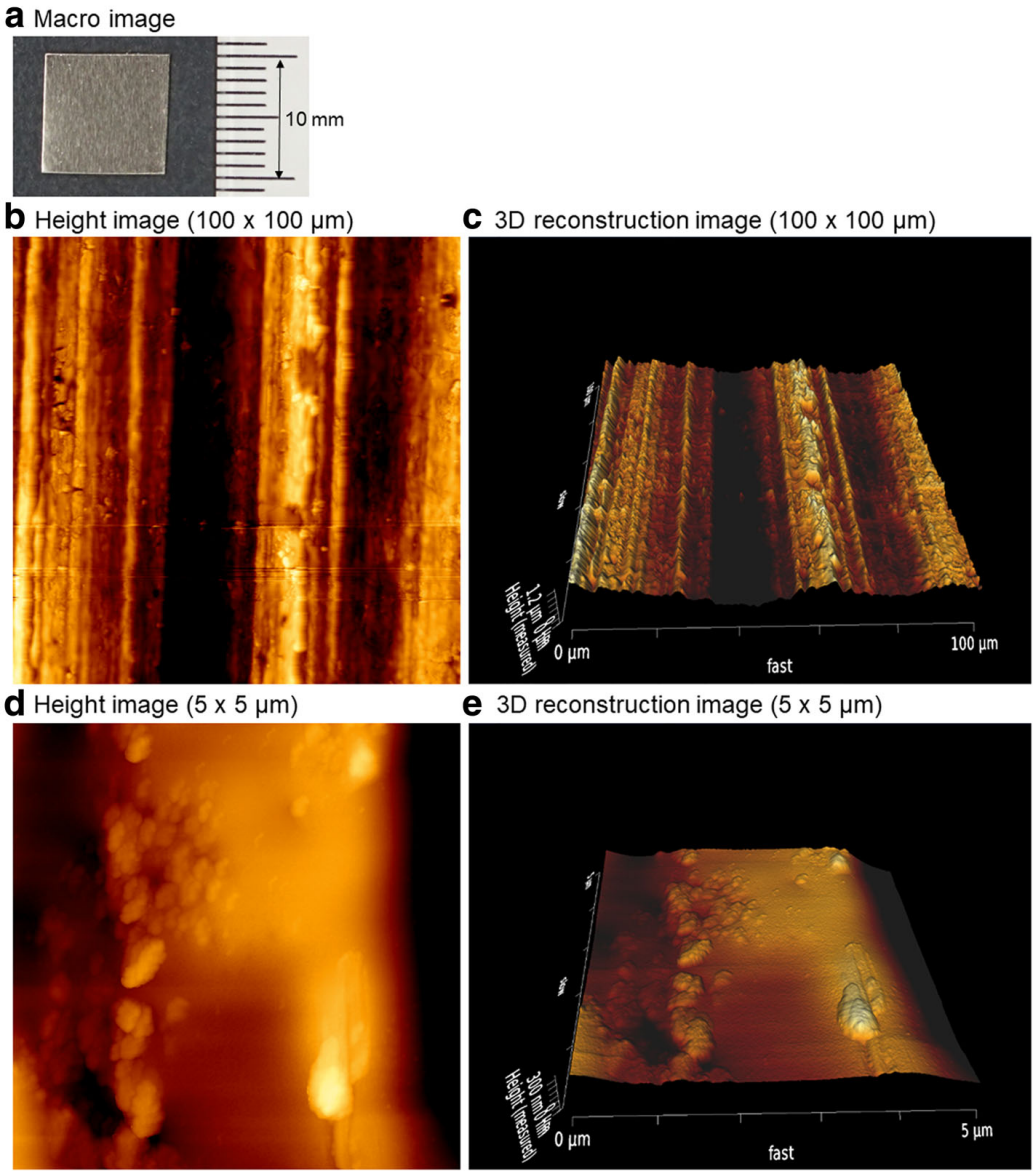
**a** Macroscopic and **b**–**e** AFM images of the plain surface of *cp*-Ti plates. **b**, **d** Height images. **c**, **e** 3D reconstruction images

Figure 1 shows the macroscopic and AFM height images of the plain surface of *cp*-Ti plates. The *cp*-Ti plates were not mirror-finished, but they looked hairline-finished macroscopically. AFM revealed the microstructure of the plain surface of the *cp*-Ti plates. At low magnification, the surface appeared to constitute narrow, straight, hairline-like grooves and ridges. However, at high magnification, small, flat spaces were observed between these microstructures.

**Fig. 2:**
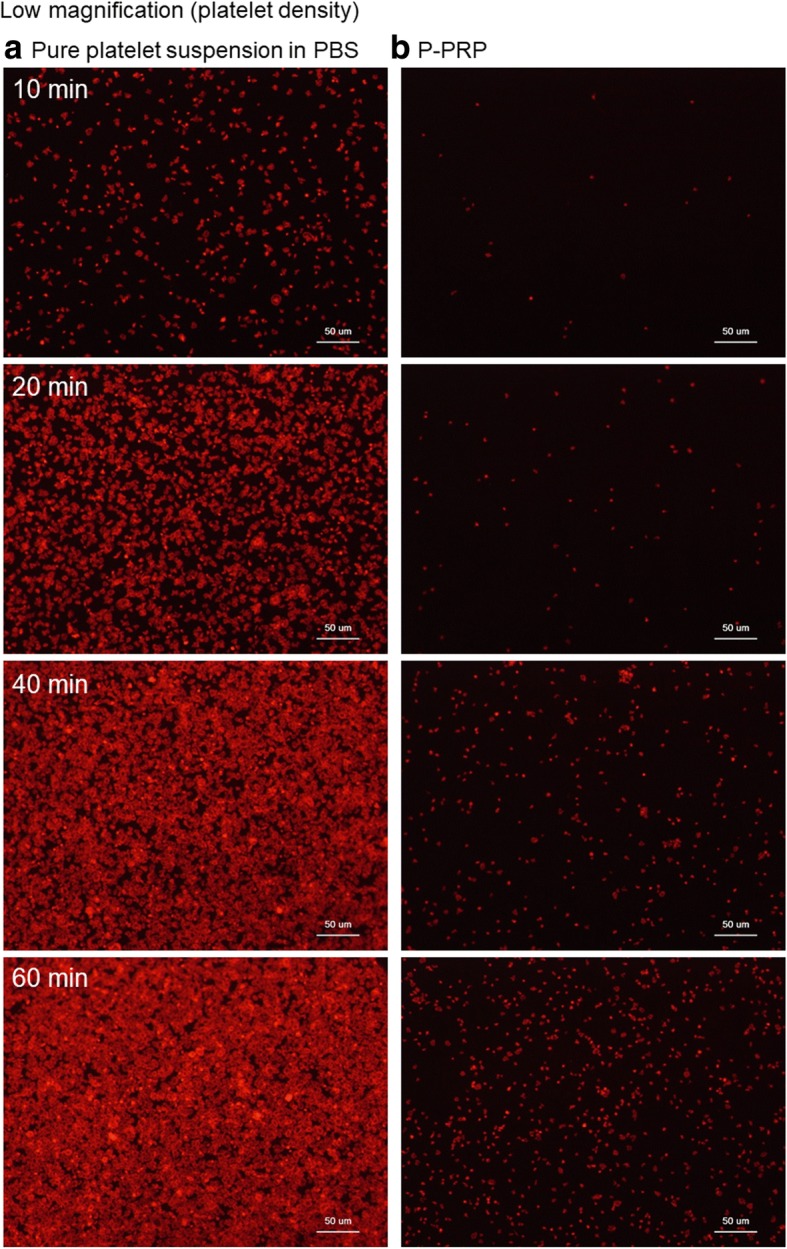
Time-course changes in platelet adhesion to the surface of *cp*-Ti plates. **a** Platelets suspended in PBS and **b** P-PRP. Actin fibers (F-actin) formed in platelets, indicating densities of platelets, are represented by a red dye. *cp*-*Ti* commercially pure titanium, *PBS* phosphate-buffered saline, *P-PRP* pure platelet-rich plasma

Figure 2 shows the time-course changes in the number of platelets adhered to the plain surface of *cp*-Ti plates. Platelets in the form of PBS suspensions adhered to the plain surface of the *cp*-Ti plates immediately after inoculation, and the number of adhered platelets increased with time, reaching maximum (i.e., confluence) adhesion at 40 min of incubation. In contrast, platelets in the form of PRP did not immediately adhere to the plain surface of the *cp*-Ti plates. In addition, although their number increased with time, it did not reach maximum adhesion within 60 min.

**Fig. 3:**
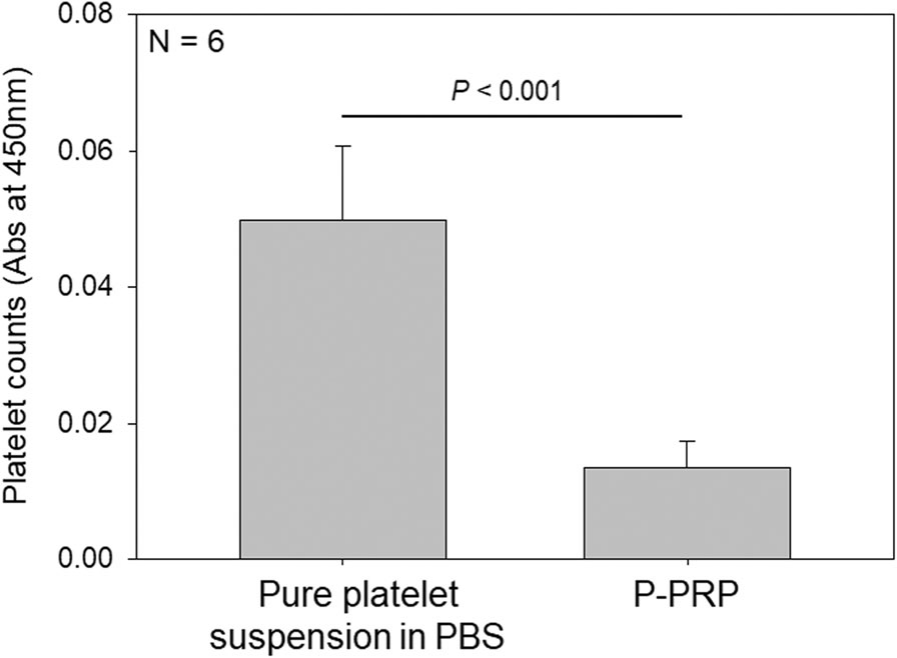
Quantitative spectrophotometric determination of platelet counts on the surface of *cp*-Ti plates

Figure 3 shows quantitative evaluations of adhered platelet counts. Platelets incubated onto the plain surface of the *cp*-Ti plates were further treated with a tetrazolium dye, and the intensity of resulting water-soluble formazan dye was spectrophotometrically analyzed. As shown in Fig. 2, platelets in the form of PBS suspensions adhered to the plain surface of the *cp*-Ti plates at a much higher extent compared with those in the form of PRP.

**Fig. 4:**
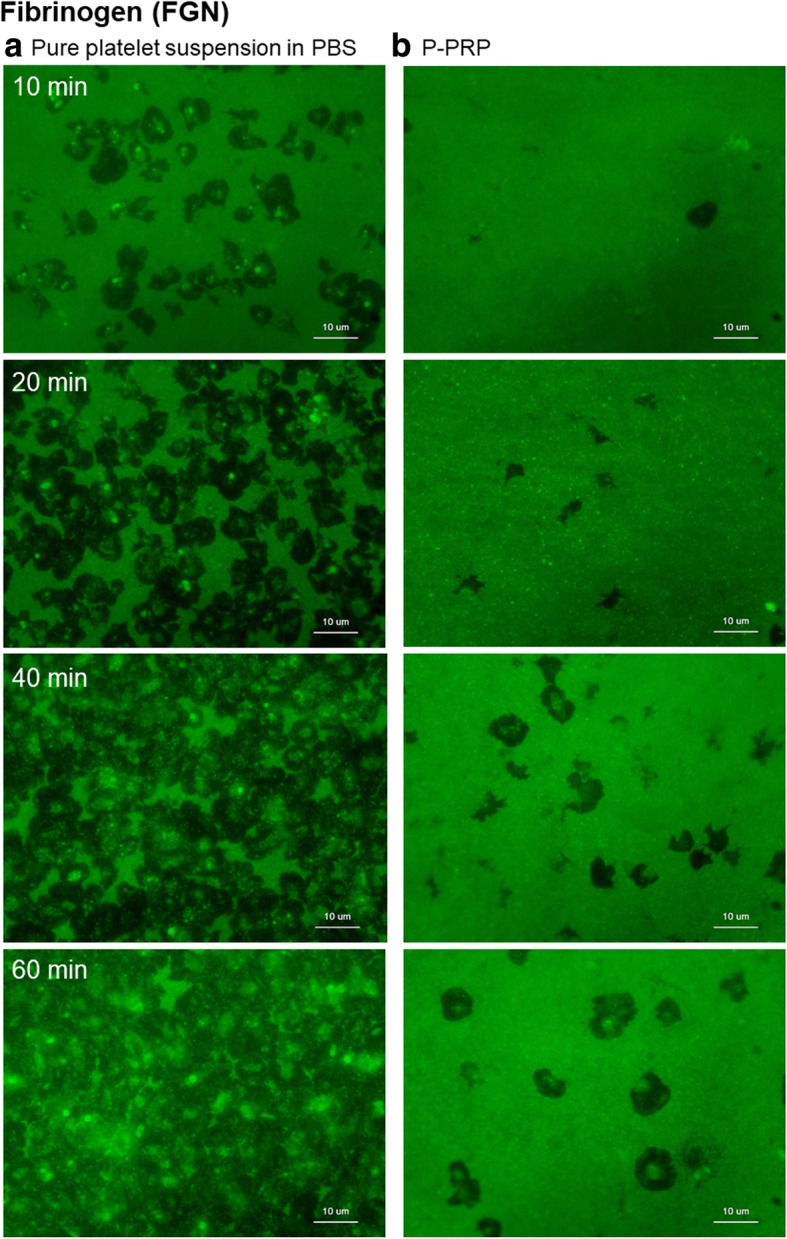
Distribution of FGN (green dye) on the surface of *cp*-Ti plates. **a** Platelets suspended in PBS and **b** P-PRP. *FGN* fibrinogen, *cp*-*Ti* commercially pure titanium, *PBS* phosphate-buffered saline, *P*-*PRP* pure platelet-rich plasma

Figure 4 shows the distribution of FGN on the plain surface of *cp*-Ti plates. In the extraplatelet space, FGN was adsorbed immediately after inoculation, and its levels were maintained for 60 min regardless of the presence or absence of plasma components. In a limited number of platelets, FGN accumulated in the platelet adhesion area, forming spots. Additional file 1: Figure S1A shows the data of negative control using isotype controls (rabbit IgG for anti-FGN, vWF, FN, albumin, and vitronectin [VN]).

**Fig. 5:**
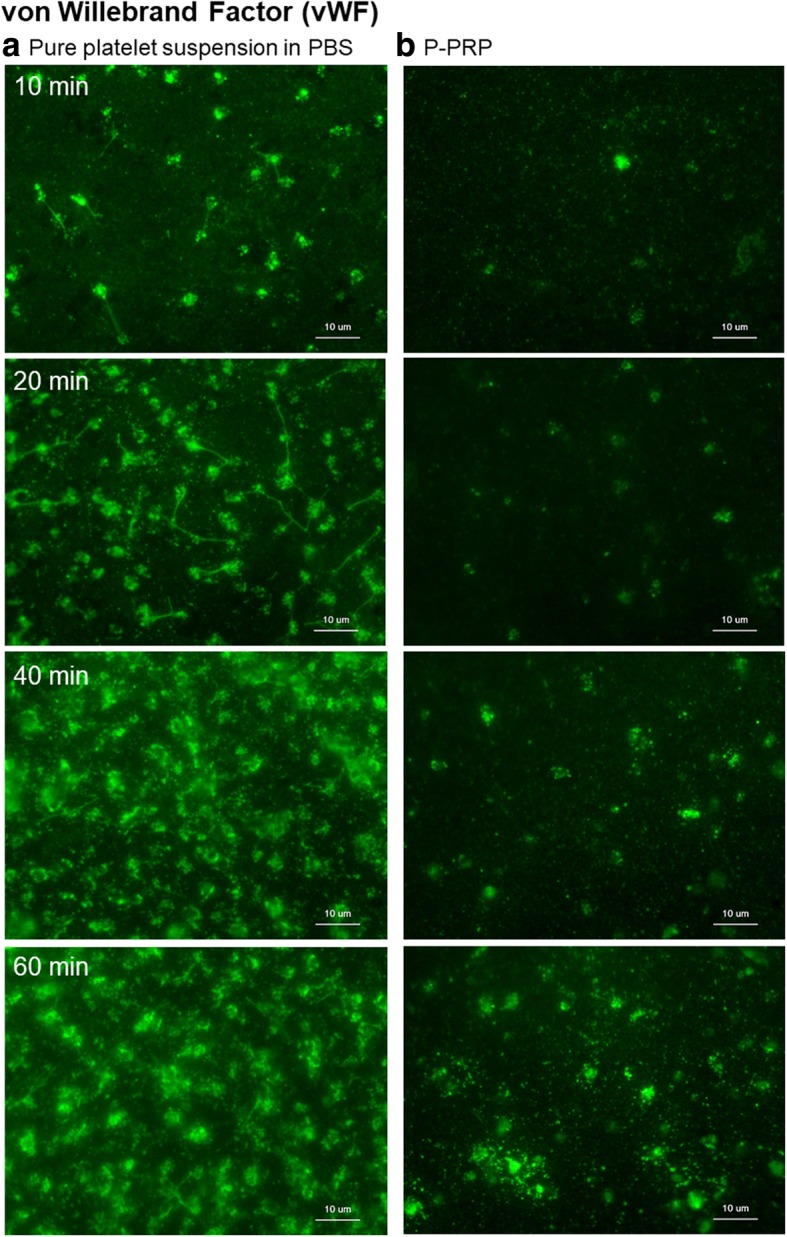
Distribution of vWF (green dye) on the surface of *cp*-Ti plates. **a** Platelets suspended in PBS and **b** P-PRP. *vWF* von Willebrand factor, *cp*-*Ti* commercially pure titanium, *PBS* phosphate-buffered saline, *P*-*PRP* pure platelet-rich plasma

Figure 5 depicts the distribution of vWF on the plain surface of *cp*-Ti plates. In the absence of plasma components, vWF immediately accumulated in the platelet adhesion area rather than in the extraplatelet space, and this characteristic distribution was maintained for up to 60 min. Similar findings were obtained in the presence of plasma components. However, compared with FGN, vWF localization in the extraplatelet space seemed disturbed by the presence of plasma components in the initial phase of aggregation, and the number of vWF-positive particles in the extraplatelet space increased with time.

**Fig. 6:**
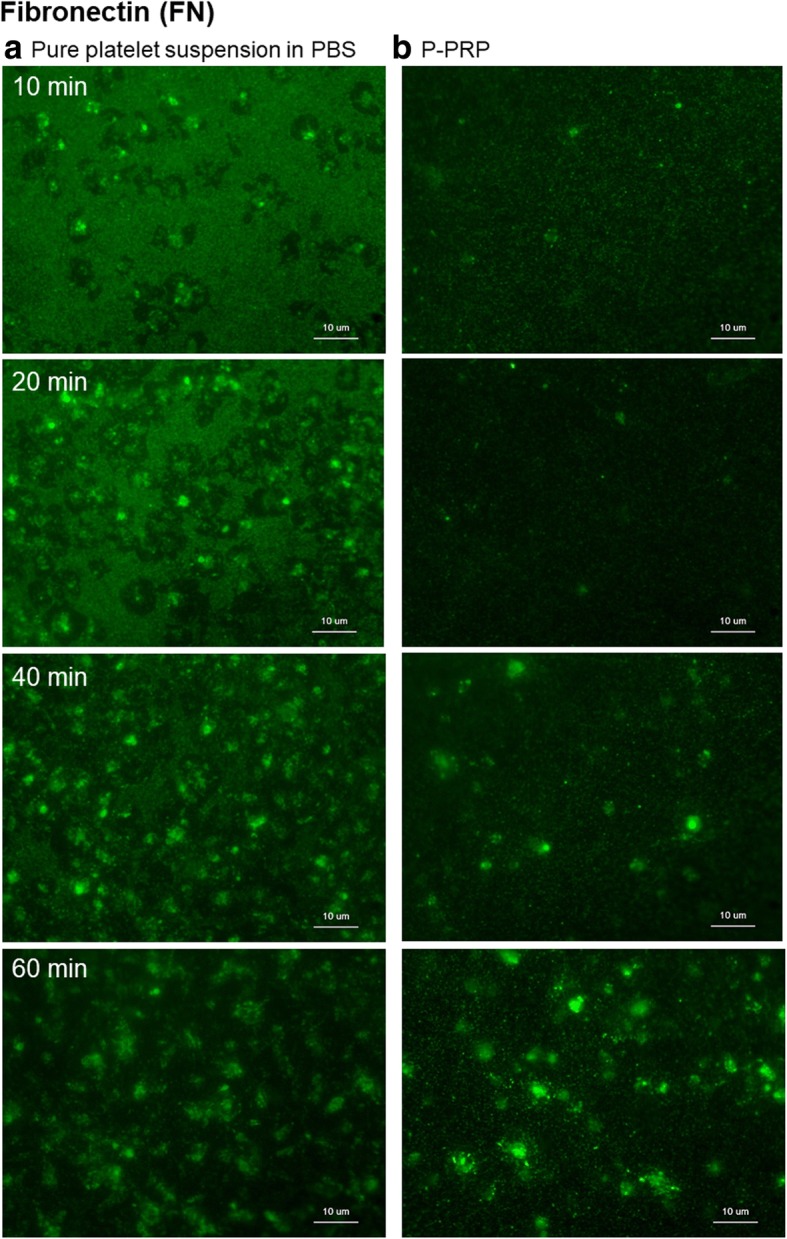
Distribution of FN (green dye) on the surface of *cp*-Ti plates. **a** Platelets suspended in PBS and **b** P-PRP. *FN* fibronectin, *cp*-*Ti* commercially pure titanium, *PBS* phosphate-buffered saline, *P*-*PRP* pure platelet-rich plasma

Figure 6 illustrates the distribution of FN on the plain surface of *cp*-Ti plates. FN distribution was somewhat between FGN and the vWF. In the absence of plasma components, FN was distributed almost equally in both the platelet adhesion area and extraplatelet space in the initial phase of aggregation, and it subsequently moved from the extraplatelet space to accumulate in the platelet adhesion area. Notably, in the presence of plasma components, FN was increasingly found both in the platelet adhesion area and extraplatelet space with time.

**Fig. 7:**
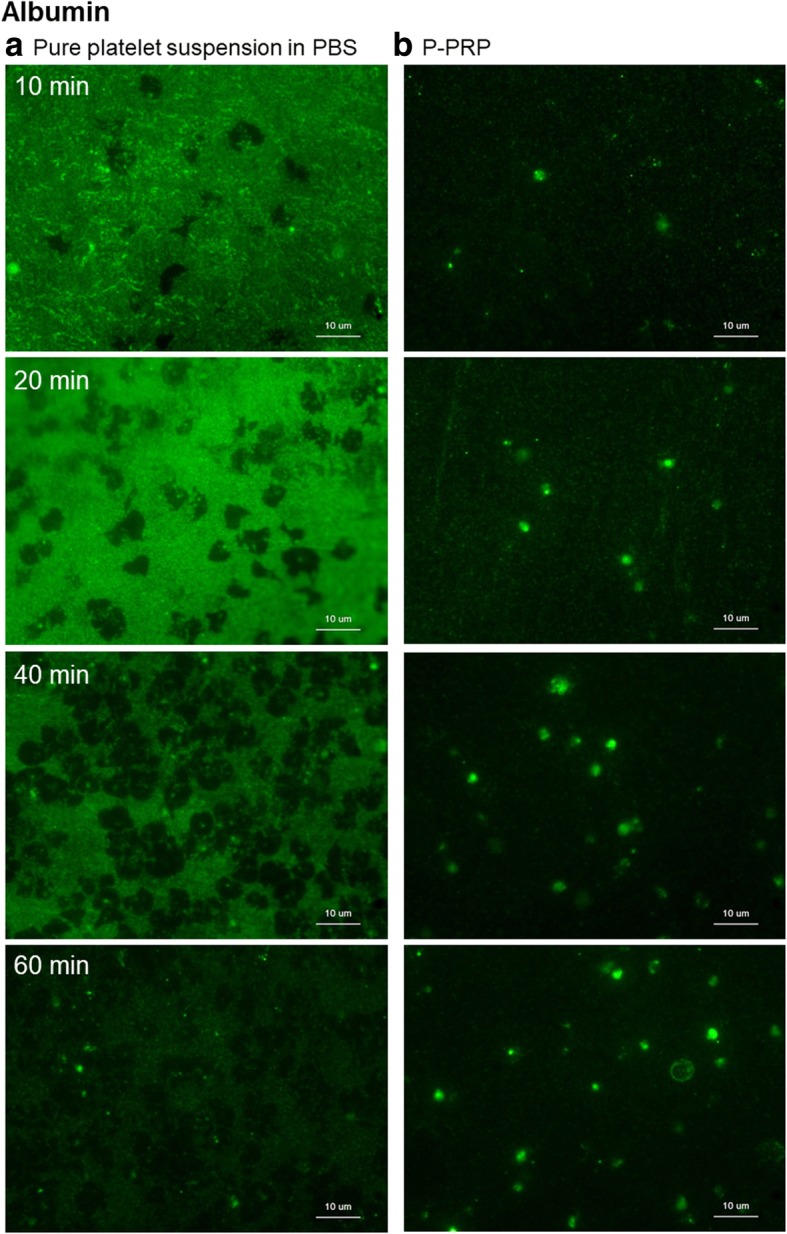
Distribution of serum albumin (green dye) on the surface of *cp*-Ti plates. **a** Platelets suspended in PBS and **b** P-PRP. *cp*-*Ti* commercially pure titanium, *PBS* phosphate-buffered saline, *P*-*PRP* pure platelet-rich plasma

Figure 7 shows the distribution of serum albumin on the plain surface of *cp*-Ti plates. In the absence of plasma components, serum albumin was rapidly adsorbed onto the plain surface of *cp*-Ti plates. In addition, as the number of adhered platelets increased, initially adsorbed serum albumin was eliminated from the platelet adhesion area, and it gradually decreased in the extraplatelet space. Compared with other platelet adhesion molecules, only a few serum albumin-positive particles were found in the platelet adhesion area. On the contrary to our prediction, in the presence of plasma components, serum albumin was hardly adsorbed onto the plain surface of the *cp*-Ti plates, but it was associated with platelets as if platelets have specific serum albumin-binding sites.

We then examined the distribution of VN on the plain surface of *cp*-Ti plates (Additional file 1: Figure S2). Despite possible differences in the antibody titers, compared with other platelet adhesion molecules, VN could not be clearly detected in our samples regardless of the presence or absence of plasma components. In general, VN was distributed in the platelet adhesion area rather than in the extraplatelet space.

**Fig. 8:**
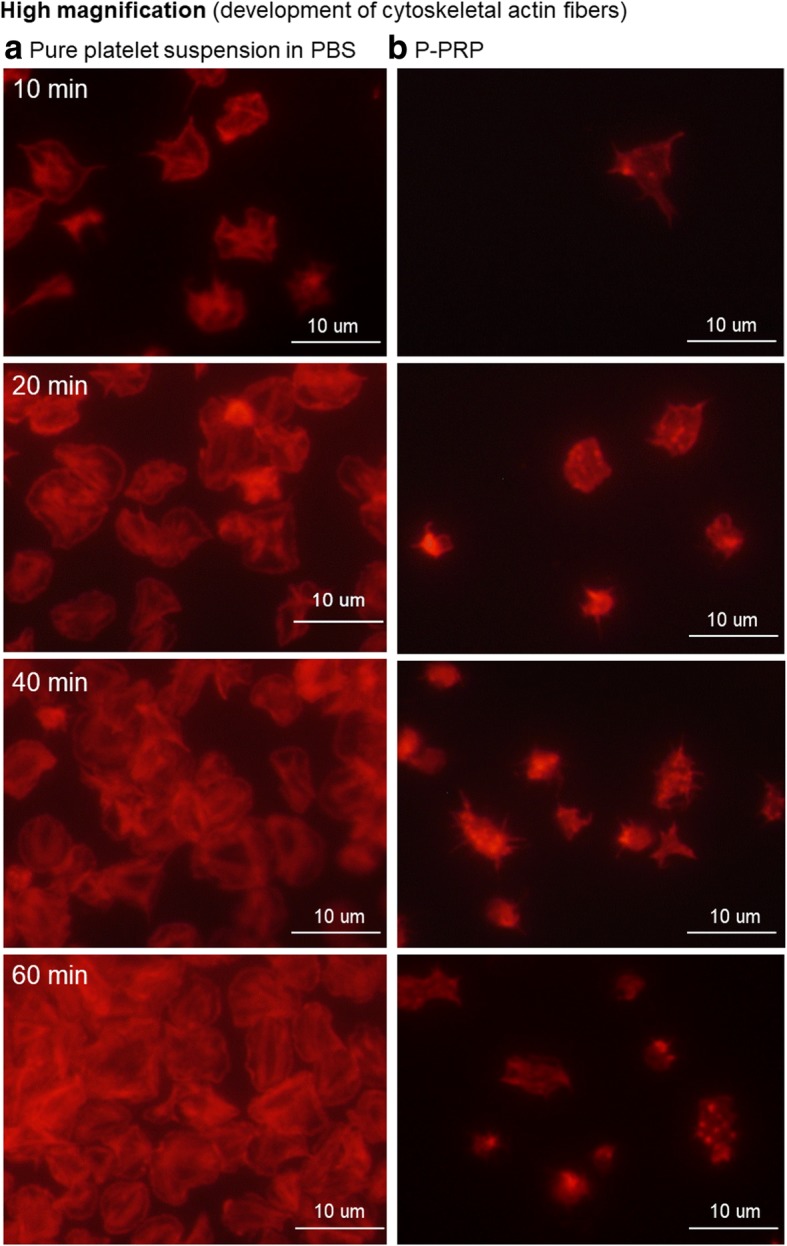
Time-course of cytoskeletal actin fiber development in adhered platelets at high magnification. **a** Platelets suspended in PBS and **b** P-PRP. *PBS* phosphate-buffered saline, *P*-*PRP* pure platelet-rich plasma

Furthermore, we examined the effects of adhesion on platelet activity. Figure 8 shows the time-course changes in cytoskeletal actin fiber development in platelets. In the absence of plasma components, sufficient development of cytoskeletal actin fibers was observed throughout the incubation period. In the presence of plasma components, however, the platelet adhesion area was substantially small and cytoskeletal actin fibers were less developed.

**Fig. 9:**
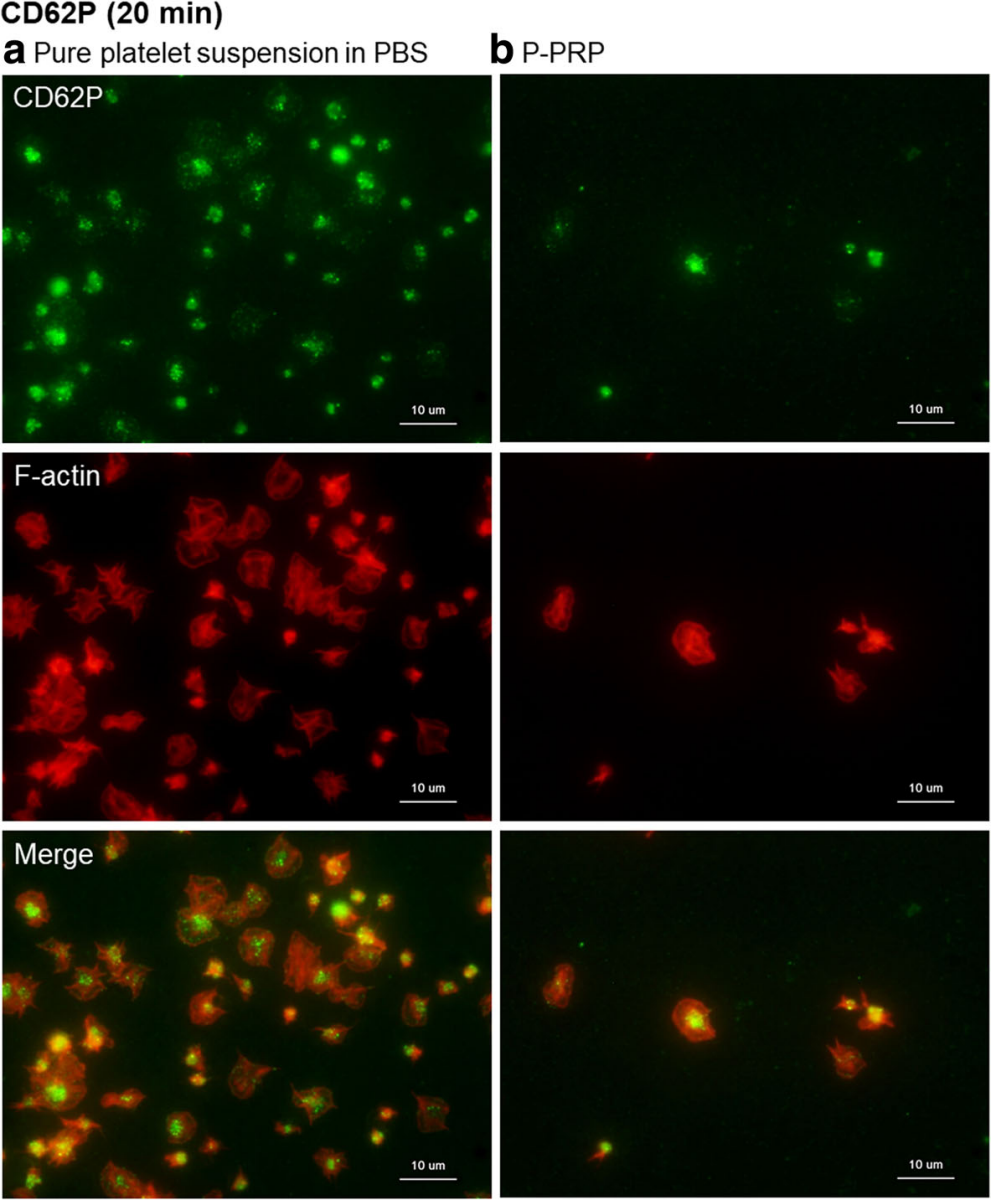
Expression of CD62P in platelets adhered onto the surface of *cp*-Ti plates. **a** Platelets suspended in PBS and **b** P-PRP. CD62P (green) and F-actin (red) were individually visualized and merged in the bottom panels. *cp*-*Ti* commercially pure titanium, *PBS* phosphate-buffered saline, *P*-*PRP* pure platelet-rich plasma

Figure 9 shows the expression of CD62P, an activated platelet marker [16, 17], in adhered platelets at 20 min of incubation. In the presence of plasma components, the platelet adhesion area and adhered platelet count were limited and granular localization of CD62P was the lowest, while in the absence of plasma components, granular localization of CD62P was observed. Additional file 1: Figure S1B shows the data of negative control using isotype controls (mouse IgG1 and κ for anti-CD62P and CD63).

**Fig. 10:**
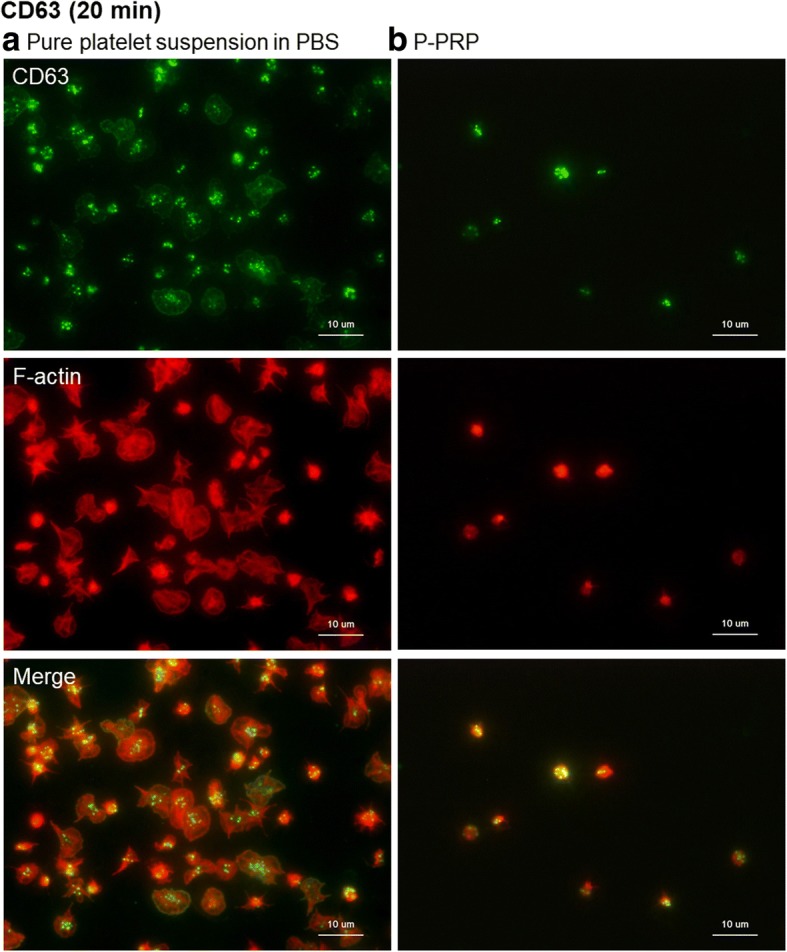
Expression of CD63 in platelets adhered onto the surface of *cp*-Ti plates. **a** Platelets suspended in PBS and **b** P-PRP. CD63 (green) and F-actin (red) were individually visualized and merged in the bottom panels. *cp*-*Ti* commercially pure titanium, *PBS* phosphate-buffered saline, *P*-*PRP* pure platelet-rich plasma

Figure 10 depicts the expression of the other activated platelet marker CD63 [16, 17] in adhered platelets. As observed with CD62P, granular localization of CD63 was somewhat higher in the absence than in the presence of plasma components.

As a positive control, time-course changes in CD63 expression in adhered platelets activated by 0.1% CaCl_2_ were evaluated (Additional file 1: Figure S3). Activated platelets showed upregulated CD62P in the initial phase of aggregation, and this elevated level was maintained for up to 60 min. On the surface of bovine serum albumin (BSA)-coated *cp*-Ti plates, CD63 expression in individual platelets seemed similar to or somewhat less than that on the surface of the control.

## Discussion

### Platelet adhesion molecules and dynamic modulation of platelet adhesion

Platelets attach and adhere to the extracellular matrix and endothelial cells or form aggregates with one another mainly through integrin αIIbβ3 (i.e., GPIIbIIIa) and GPIb-V-IX (receptors) during thrombus formation although platelets express various receptors for platelet adhesion molecules [17–19]. In the initial phase of aggregation, resting discoid platelets attach to flat substrates (e.g., vascular endothelial cells in vivo) with filopodia through FGN–integrin αIIbβ3 binding [20]. In the subsequent growing phase, in addition to integrin αIIbβ3, GPIb-V-IX is involved in aggregation through vWF, FGN, and FN. In the final phase, GPIb-V-IX acts as a predominant adhesion receptor to form rolling aggregates with vWF. These findings indicate that FN and vWF play crucial roles in the initial and late phases of aggregation, respectively.

**Fig. 11:**
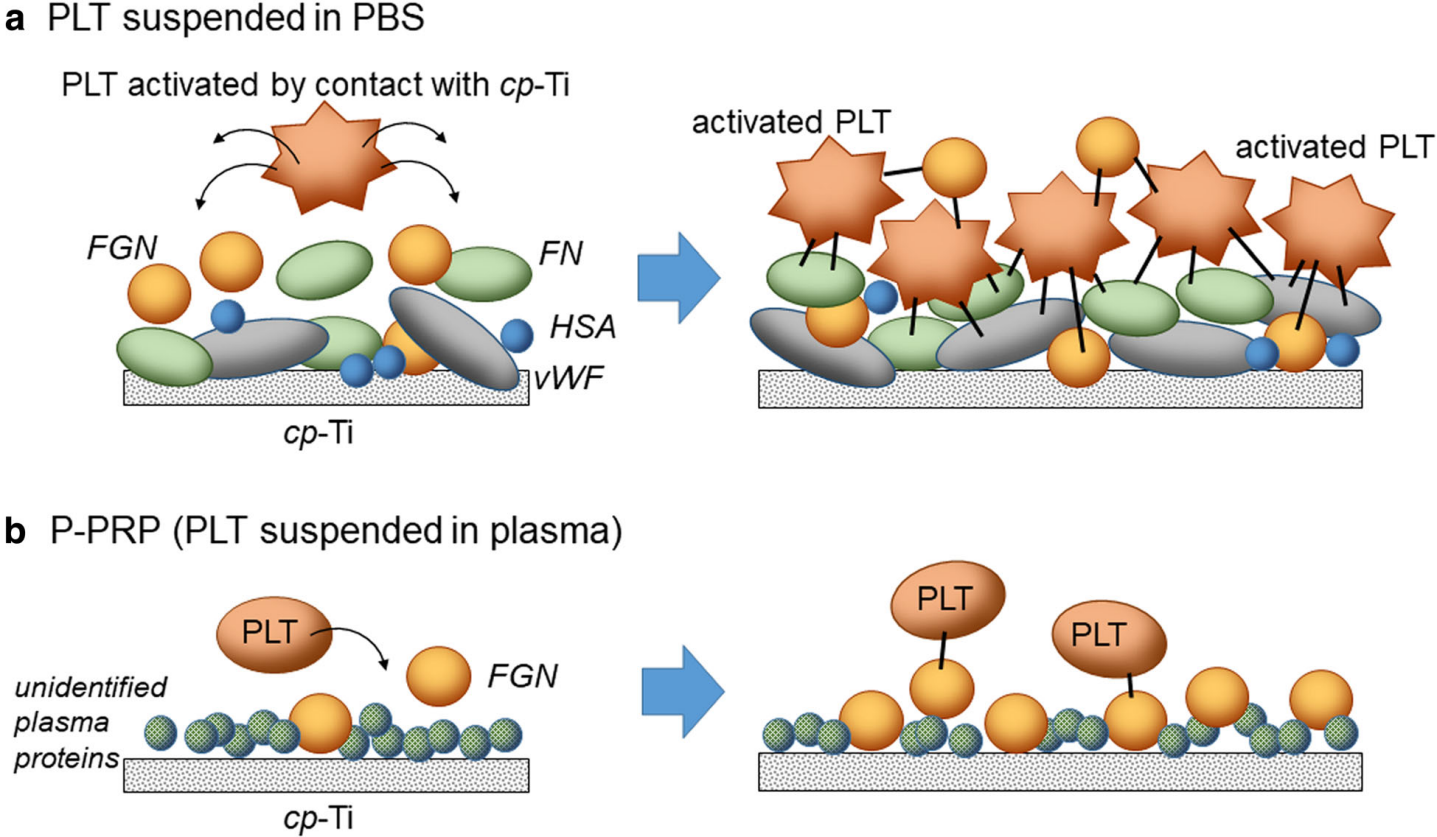
Possible mechanisms of protein adsorption and platelet adhesion on *cp*-Ti plates. **a** Platelets suspended in PBS and **b** P-PRP. (Left and center) Time-course changes in protein adsorption and platelet adhesion. (Right) Possible relationships between adhesion molecules and their receptors expressed in platelets. *cp*-*Ti* commercially pure titanium, *PBS* phosphate-buffered saline, *P*-*PRP* pure platelet-rich plasma, PLT platelet

On the basis of this scenario of in vivo clot formation, possible mechanisms of protein adsorption and platelet adhesion on *cp*-Ti plates are illustrated in Fig. 11. We believe that in the absence of plasma components (unidentified in this study), platelets can be easily activated by contact with *cp*-Ti plates to release adhesion molecules (e.g., FGN, vWF, and FN), which can be predominantly adsorbed onto the surface of *cp*-Ti plates, and adhere to the plates through these adhesion molecules (for more details, see the next subsection). Platelets have two major types of adhesion systems (i.e., integrin αIIbβ3 and GPIb-V-IX) [20]; therefore, in the absence of plasma components, these activated platelets are believed to bind to *cp*-Ti plates more tightly compared to less activated platelets.

### Association between plasma components and platelet adhesion molecules

Platelets accumulate at sites of injury to prevent bleeding and release growth factors to repair damaged tissue and organs in vivo. Under in vitro conditions, a series of these reactions could be modified to some extent against foreign substrates, particularly in the presence of anticoagulants that deplete Ca^2+^ from coagulation cascades and platelets to prevent platelet activation.

In this study, we observed that platelet adhesion to the plain surface of *cp*-Ti plates was disturbed in the presence of plasma components. Similarly, the adsorption of other platelet adhesion molecules, except FGN, was disturbed in the presence of plasma components. In contrast, in the absence of plasma components, platelets time-dependently adhered to the surface of *cp*-Ti plates with well-developed cytoskeletal actin fibers, reaching the maximum adhesion at 40 min of incubation. In addition, in the absence of plasma components (or, more precisely, in the presence of reduced plasma components), vWF, FGN, and FN were immediately adsorbed on the surface of *cp*-Ti plates.

Taken together, these findings suggest that among platelet adhesion molecules, vWF and FN were favored for platelet adhesion compared with FGN and VN. VN, if present in the platelet suspensions, was not absorbed on *cp*-Ti plate surface or used for adhesion as much as the other platelet adhesion molecules.

### Possible mechanisms underlying the blocking action of serum albumin

In the presence of plasma components, adsorption of platelet adhesion molecules (except FGN) and adhesion of platelets were similarly blocked. Serum albumin is the most abundant plasma protein in humans and several other animals, and BSA [21, 22] is often used as a blocking agent to prevent unspecific protein adsorption onto the surface of devices for handling biological samples [21–23]. However, in this study, although perplexed, albumin was much less visualized on the surface of P-PRP-treated *cp*-Ti plates compared to the control surface. This finding suggested two possibilities: (i) serum albumin might not necessarily interfere with platelet adhesion, but, with time, can be replaced gradually with other adhesion molecules, and (ii) serum albumin can be excluded by unidentified plasma components.

Regarding the former possibility, serum albumin lacks any known amino acid sequences or binding protein receptors; therefore, this abundant serum protein cannot support platelet adhesion or aggregation [24, 25]. However, Sivaraman and Lautour [24] proposed that in its adsorption state, serum albumin changes the structure of a large number of charged amino acids (i.e., 24 arginine, 59 lysine, 35 aspartic acid, and 62 glutamic acid residues) recognized by Arg-Gly-Asp (RGD)-specific receptors in platelets similar to an RGD motif. In addition, studies have reported that serum albumin shows significant anticoagulant action in a concentration-dependent manner [26]. According to this theory, serum albumin that remained in platelet pellets produced by centrifugation might be able to support initial platelet adhesion in collaboration with other genuine adhesion molecules. At the very least, it might not be surprising that at low concentrations, serum albumin does not interfere with platelet adhesion under these artificial conditions.

Regarding the latter possibility, we initially hypothesized that PRP-containing serum albumin, whose concentration is much higher than in a platelet suspension in PBS, is immediately adsorbed onto the surface of *cp*-Ti plates, blocking platelet adhesion. As expected, in this study, platelet adhesion and vWF and FN adsorption were blocked in the presence of plasma components. However, we did not observe significant levels of albumin adsorbed onto the surface of PRP-treated *cp*-Ti plates. This finding suggested that other unidentified plasma components preferentially and rapidly bind to *cp*-Ti plates and reduce the space for albumin adsorption. Otherwise, although albumin is initially adsorbed onto the surface, it can be immediately replaced by other unidentified plasma components. This phenomenon is called the Vroman effect [27]. Although studies have reported that when introduced as the second protein, BSA displaces already existing larger proteins (e.g., FGN and FN) from the surface of *cp*-Ti plates [28],; and in this study, unidentified plasma components like smaller proteins probably displaced the albumin initially adsorbed onto the *cp*-Ti plates. Further studies are required in order to identify these plasma components and elucidate this phenomenon.

### Exceptional adsorption of FGN in the presence of plasma components

Why adsorption of FGN, but not the other adhesion molecules, was not inhibited by plasma components, although the *cp*-Ti surface was not covered by albumin remains unknown. FGN was not adsorbed on the surface of BSA-coated *cp*-Ti plates (Additional file 1: Figure S4). Interestingly, adsorbed FGN (and vWF) can promote platelet adhesion more potently than FN [29], and it is adsorbed on the top of serum albumin in a bilayer or multilayer [23, 28]. Moreover, FGN stimulates platelet adhesion after being adsorbed onto a surface but not in its native form (nonadsorbed) [30], and it associates with TiO_2_ markedly faster than human serum albumin [31].

In this study, in the absence of plasma components, platelet adhesion molecules were adsorbed together with serum albumin on the surface of *cp*-Ti plates, indicating that these proteins are accumulated on the surface in a multilayer. FGN, but not vWF or FN, in the upper layer may reduce its ability to adhere to platelets.

### Platelet activation

Integrins function as dynamic scaffolds, which facilitate platelet adhesion onto surrounding cells or artificial surfaces, and as receptors for external signaling molecules, such as FGN, vWF, and FN [19, 24, 30, 32–36]. In this study, we examined platelet activation by the development of cytoskeletal actin fibers and the expression of surface markers. Regardless of the number of adhered platelets, F-actin formation was well developed in adhered platelets from earlier phases in the absence of plasma components but poorly developed or absent in the presence of plasma components. Similarly, both CD62P and CD63 were upregulated in a time-dependent manner in the absence of plasma components. These results suggest that plasma components interfere with the adhesion of floating platelets and subsequent activation of adhered platelets.

### Possible biomedical significance and clinical relevance

Finally, the biomedical significance of dental implant pretreatment with P-PRP prior to implantation should be briefly discussed. In this study, we observed that the adhesion and adsorption of platelets and major platelet adhesion molecules on the plain surface of *cp*-Ti plates were disturbed in the presence of plasma components. Although we observed that prolonged P-PRP treatment increases platelet adhesion, our findings do not support the theory that rapid pretreatment with P-PRP may positively functionalize the plain surface of *cp*-Ti plates.

Some clinical and preclinical animal studies have supported the use of PRP for better dental implant stability or osseointegration [3, 6, 7, 10, 37]. However, other studies have reported no positive effects [4, 5, 8, 9]. Recently developed dental implants have surfaces modified mechanically or (electro) chemically to improve their biocompatibility and tissue integration. The microrough surfaces of dental implants might be suitable to efficiently trap platelets, platelet adhesion molecules, and growth factors such that the dental implant surface is positively functionalized even with rapid pretreatment.

This controversial situation seems identical to that of skeletal regeneration using PRP [38]. PRP is considered an adjuvant therapy, and it largely depends on successful angiogenesis [2, 39]. Although growth factors and platelet adhesion molecules can be adsorbed more easily onto the modified surface, e.g., a microrough surface, of dental implants and retained more efficiently, effects which are expected to initially work for better cell adhesion and growth, to our knowledge, there is no convincing evidence that supports better subsequent stabilization and consequent osseointegration.

This lack of evidence might be due to variations in application protocols, differences in bone conditions at the site of implantation, and variations in individual PRP quality. In this study, we examined platelet adhesion under the most popular protocol for PRP application—coating by simple immersion. This procedure is superior in terms of complete exclusion of air bubbles generated on the implant surface by visual inspection. To our knowledge, no studies have exclusively performed direct application of PRP in any form [40] or platelet-rich fibrin (PRF) in implant holes to improve the initial stability of implants. When implant hole conditions are not favorable for mechanically tight contact between bone and implant surface, a clotted PRP or PRF membrane might function like a sealing tape to temporally and mechanically stabilize the implants. However, it is not yet accepted that the functionalization of implants using PRP, like the PRP immersion procedure, contributes to stabilization of placed implants in the medium term.

On the other hand, when bone quality is high, the bone is stiff enough to retain implants tightly and flexibly without the occurrence of a gap during rotation with frictional force. Under these conditions, relatively larger components (e.g., platelets and fibrin) coated on the implant surface are probably easily removed during rotation. Therefore, PRP might not appreciably contribute to further improvement of implant stability, but might somewhat contribute to the improvement of implant bioaffinity by securing platelet-derived small molecules and excluding air bubbles.

Compared to other factors described earlier, we believe that PRP quality is usually a more crucial factor for better clinical outcomes. As described in previous studies, we recommend that clinical implantologists use high-quality PRP preparations for surface modification of implants as in other regenerative therapies [41, 42].

## Conclusions

Platelets mainly use vWF and FN for adhesion onto the surface of *cp*-Ti plates. However, this adhesion is substantially disturbed by the presence of plasma nonadhesion molecules. Although the data obtained in this study are limited, therefore, titanium dental implants with a plain surface, such as machined implants, may not be significantly functionalized by a rapid PRP pretreatment.

## Additional file

Additional file 1: **Figure S1.** Negative controls for immunofluorescent visualization of FGN, vWF, FN, CD62P, and CD63. Control platelets incubated on *cp*-Ti plates for 20 mins were used. FGN, fibrinogen; vWF, von Willebrand factor; FN, fibronectin; *cp*-Ti, commercially pure titanium. Figure S2. Negative controls for immunofluorescent visualization of FGN, vWF, FN, CD62P, and CD63. Control platelets incubated on *cp*-Ti plates for 20 mins were used. FGN, fibrinogen; vWF, von Willebrand factor; FN, fibronectin; *cp*-Ti, commercially pure titanium. Figure S3. Time-course changes in adhesion of CD63^+^ platelets. (A) Platelets suspended in PBS without activation and (B) 0.1% CaCl2-activated platelets suspended in PBS. (Left bottom) Control platelets incubated on BSA-coated *cp*-Ti plates for 20 mins. PBS, phosphate-buffered saline; BSA, bovine serum albumin; *cp*-Ti, commercially pure titanium. Figure S4. Adsorption of FGN, vWF, FN, and VN onto the surface of BSA-coated *cp*-Ti plates. Platelets suspended in PBS on the (A) control surface and (B) BSA-coated surface. FGN, fibrinogen; vWF, von Willebrand factor; FN, fibronectin; VN, vitronectin; *cp*-Ti, commercially pure titanium; PBS, phosphate-buffered saline; BSA, bovine serum albumin. (DOCX 2495 kb)

## Abbreviations

ACD-A: A formulation of acid-citrate-dextrose solution (new line) AC Alternating current
AFM: Atomic force microscope
BSA: Bovine serum albumin (new line) *cp*-Ti commercially pure titanium
FGN: Fibrinogen
FN: Fibronectin
HSA: Human serum albumin
PGE_1_: Prostaglandin E_1_
PRP: Platelet-rich plasma (new line) P-PRP pure PRP
SD: Standard deviation
T-PBS: 0.1% Tween-20-containing PBS
VN: Vitronectin
vWF: von Willebrand factor
