# Supplementary material for: Platelet adhesion on commercially pure titanium plates in vitro I: effects of plasma components and involvement of the von Willebrand factor and fibronectin
Source: Int J Implant Dent. 2019 Feb 25;5:5. doi: 10.1186/s40729-019-0160-z (PMC6387980; doi:10.1186/s40729-019-0160-z)
Supplement: Supplementary file 1 — Figure S1. Negative controls for immunofluorescent visualization of FGN, vWF, FN, CD62P, and CD63. Control platelets incubated on cp-Ti plates for 20 mins were used. FGN, fibrinogen; vWF, von Willebrand factor; FN, fibronectin; cp-Ti, commercially pure titanium. Figure S2. Negative controls for immunofluorescent visualization of FGN, vWF, FN, CD62P, and CD63. Control platelets incubated on cp-Ti plates for 20 mins were used. FGN, fibrinogen; vWF, von Willebrand factor; FN, fibronectin; cp-Ti, commercially pure titanium. Figure S3. Time-course changes in adhesion of CD63+ platelets. (A) Platelets suspended in PBS without activation and (B) 0.1% CaCl2-activated platelets suspended in PBS. (Left bottom) Control platelets incubated on BSA-coated cp-Ti plates for 20 mins. PBS, phosphate-buffered saline; BSA, bovine serum albumin; cp-Ti, commercially pure titanium. Figure S4. Adsorption of FGN, vWF, FN, and VN onto the surface of BSA-coated cp-Ti plates. Platelets suspended in PBS on the (A) control surface and (B) BSA-coated surface. FGN, fibrinogen; vWF, von Willebrand factor; FN, fibronectin; VN, vitronectin; cp-Ti, commercially pure titanium; PBS, phosphate-buffered saline; BSA, bovine serum albumin. (DOCX 2495 kb) [file 40729_2019_160_MOESM1_ESM.docx]

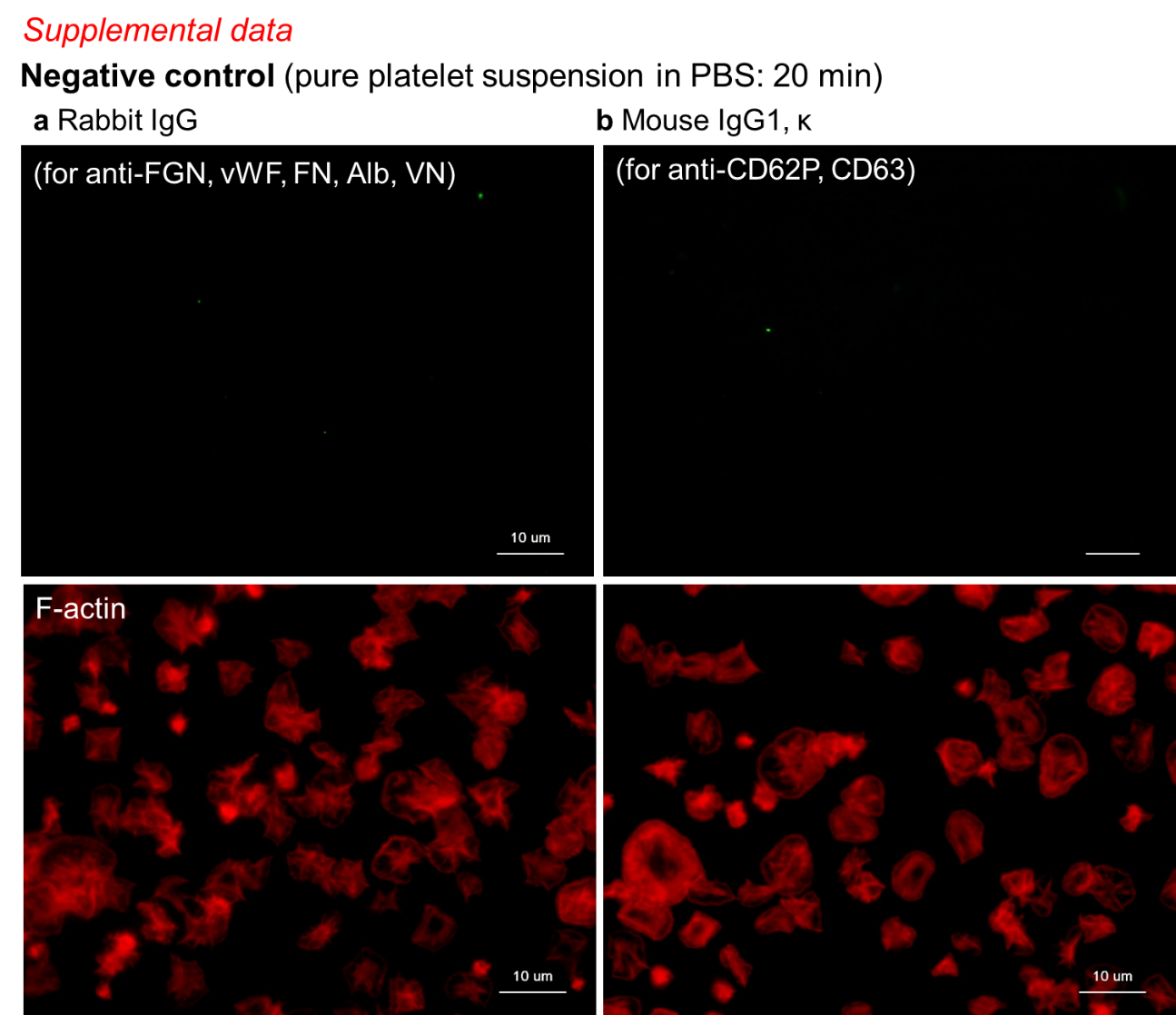


**Fig. S1** Negative controls for immunofluorescent visualization of FGN, vWF, FN, CD62P, and CD63. **a** An isotype control for Rabbit IgG, **b** an isotype control for Mouse IgG1, κ. Control platelets incubated on *cp*-Ti plates for 20 mins were used. FGN, fibrinogen; vWF, von Willebrand factor; FN, fibronectin; *cp*-Ti, commercially pure titanium.


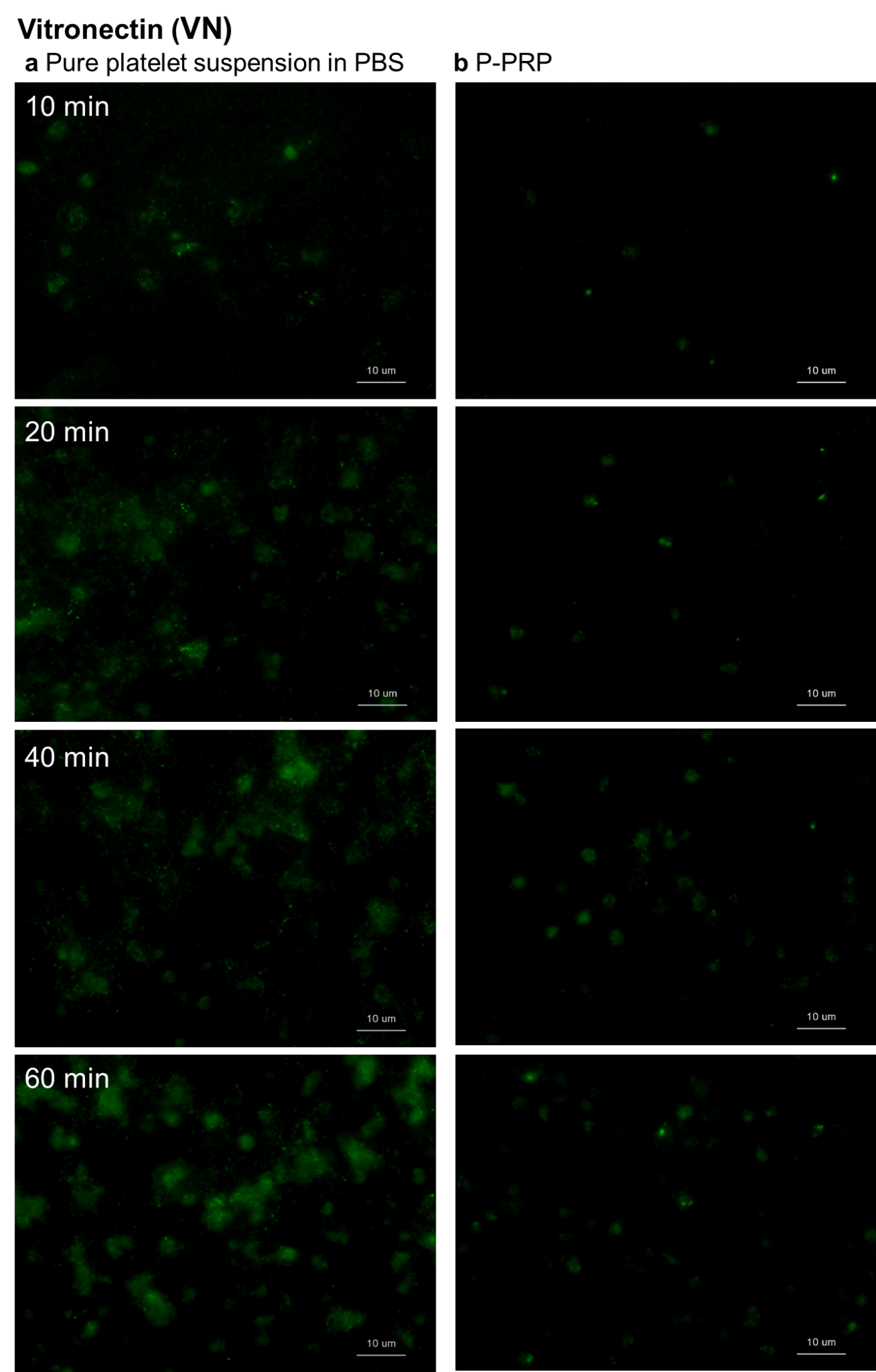


**Fig. S2** Negative controls for immunofluorescent visualization of FGN, vWF, FN, CD62P, and CD63. Control platelets incubated on *cp*-Ti plates for 20 mins were used. **a** Platelets suspended in PBS and **b** PRP. FGN, fibrinogen; vWF, von Willebrand factor; FN, fibronectin; *cp*-Ti, commercially pure titanium.


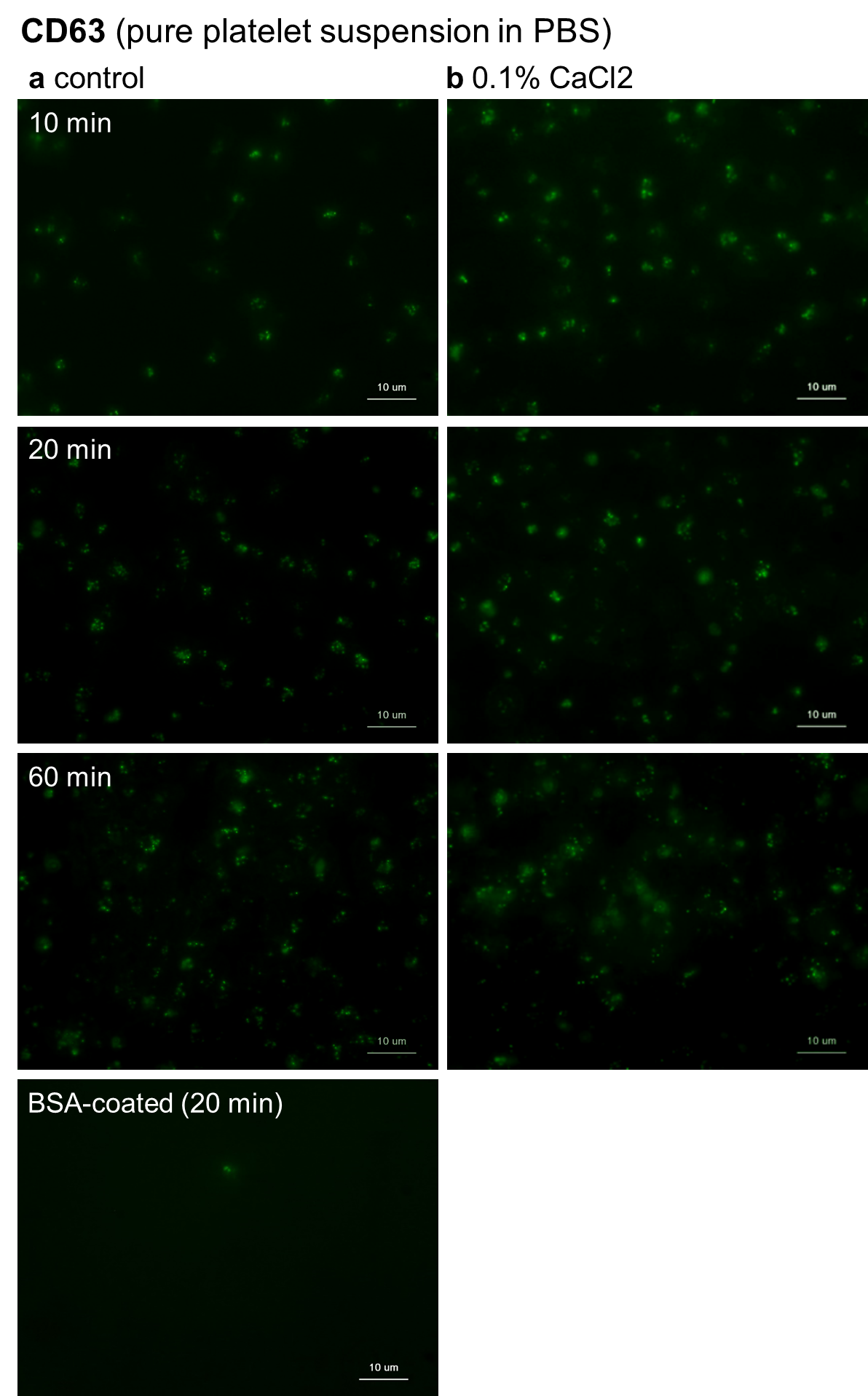


**Fig. S3** Time-course changes in adhesion of CD63^+^ platelets. **a** Platelets suspended in PBS without activation and **b** 0.1% CaCl2-activated platelets suspended in PBS. (Left bottom) Control platelets incubated on BSA-coated *cp*-Ti plates for 20 mins. PBS, phosphate-buffered saline; BSA, bovine serum albumin; *cp*-Ti, commercially pure titanium.


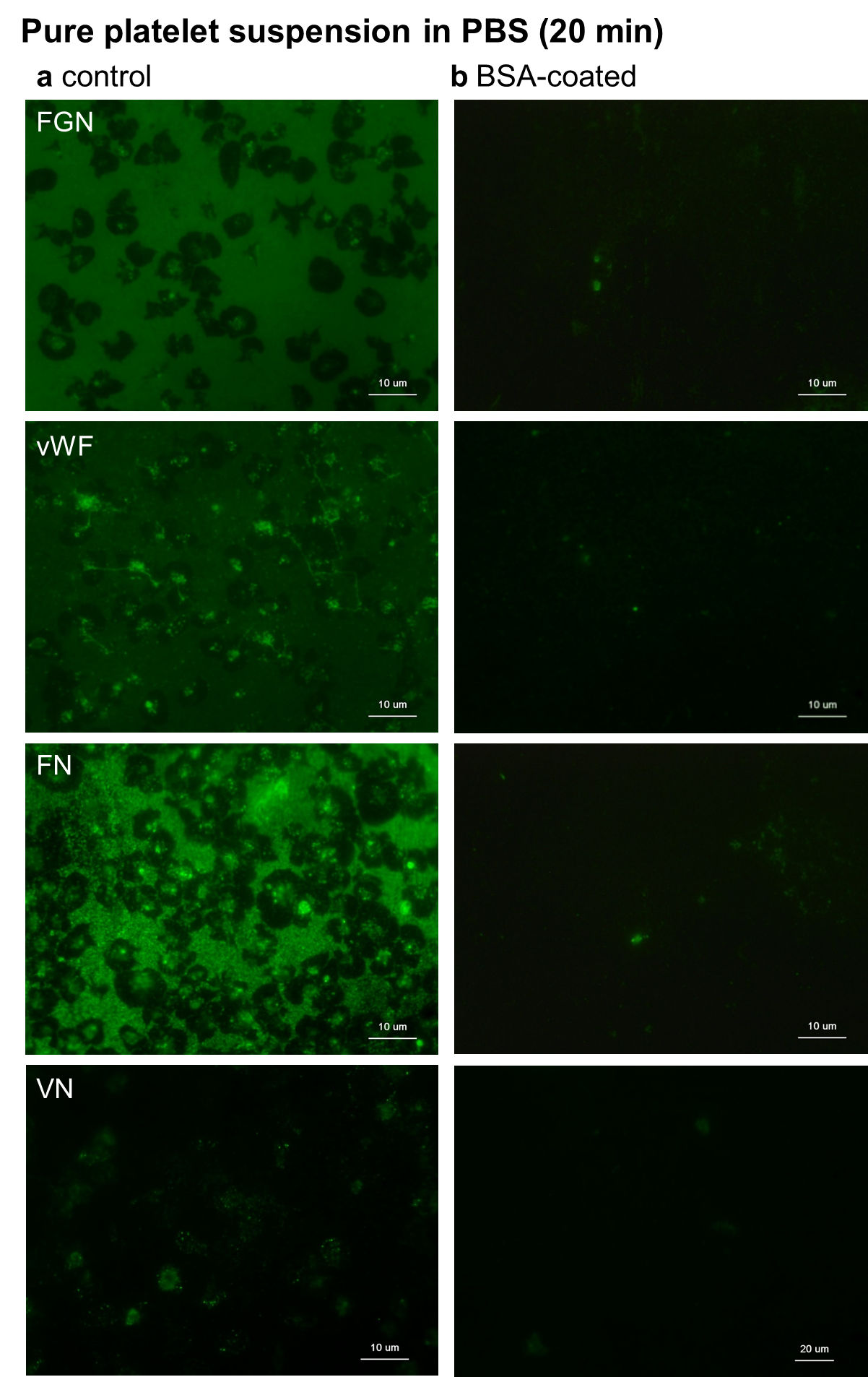


**Fig. S4** Adsorption of FGN, vWF, FN, and VN onto the surface of BSA-coated *cp*-Ti plates. Platelets suspended in PBS on **a** the control surface and **b** BSA-coated surface. FGN, fibrinogen; vWF, von Willebrand factor; FN, fibronectin; VN, vitronectin; *cp*-Ti, commercially pure titanium; PBS, phosphate-buffered saline; BSA, bovine serum albumin.
